# Optical coherence tomography catheter-induced vasospasm of the left anterior descending artery: A case report

**DOI:** 10.1016/j.radcr.2024.07.035

**Published:** 2024-08-07

**Authors:** Fawaz Mohammed, Zaina Ali Khan, Muna Mohammed, Evan D. Gleaves, Cody Schwartz, Sajjad Haider, Sameer Saleem, Akhtar Amin, Jacqueline Dawson Dowe, Mohammed Abdul-Waheed, Muhammad Shoaib Akbar, Mohammed Kazimuddin, Rahil Rafeedheen

**Affiliations:** a Department of Medicine, University of Kentucky, Bowling Green, KY 42101, USA; b Deccan College of Medical Sciences, Hyderabad, Telangana 50008, India; c Department of Cardiology, University of Kentucky, Bowling Green, KY 42101, USA; d Department of Cardiology, Med Center Health, Bowling Green, KY 42101, USA

**Keywords:** Optical coherence tomography, Coronary artery vasospasm, Coronary angiography

## Abstract

Optical coherence tomography (OCT) helps identify coronary artery disease of different etiologies. Vasospasm from OCT catheter is a rarely reported complication that is more commonly seen in the right coronary artery. We report a case of OCT-catheter induced vasospasm of the left anterior descending artery that resolved with administration of nitroglycerine. Interventionalists need to weary of the occurrence of catheter-related coronary artery spasm to avoid stenting when not necessary.

## Introduction

Optical coherence tomography (OCT) is used to identify and differentiate angiographic lesions in the coronary arteries from various phenotypes of coronary artery disease [1]. Coronary vasospasm is a rare complication of left heart catheterization, with an estimated occurrence of 0.75% [2]. More commonly this adverse event is seen in the right coronary artery although involvement of the left coronary arteries has been reported in the literature [3]. Spasms may involve multiple areas of a solitary coronary artery although cases of more than one artery being involved have also been described [3]. Catheter-induced coronary artery vasospasm is most discussed in the context of case reports; however, one retrospective study demonstrated an occurrence of 0.75% in the right coronary artery (RCA) [[2], [3], [4]]. In the setting of atherosclerotic disease, the diagnosis of catheter-induced coronary spams is often challenging, and interventionalists need to be cautious to mitigate risks from cardiovascular complications in diseased coronary arteries and avoid coronary revascularization in cases when not necessary [5]. Herein, we report a case of introduction of the OCT catheter inducing coronary vasospasm that resolved with administration of intracoronary nitroglycerine.

## Case presentation

**Fig. 1:**
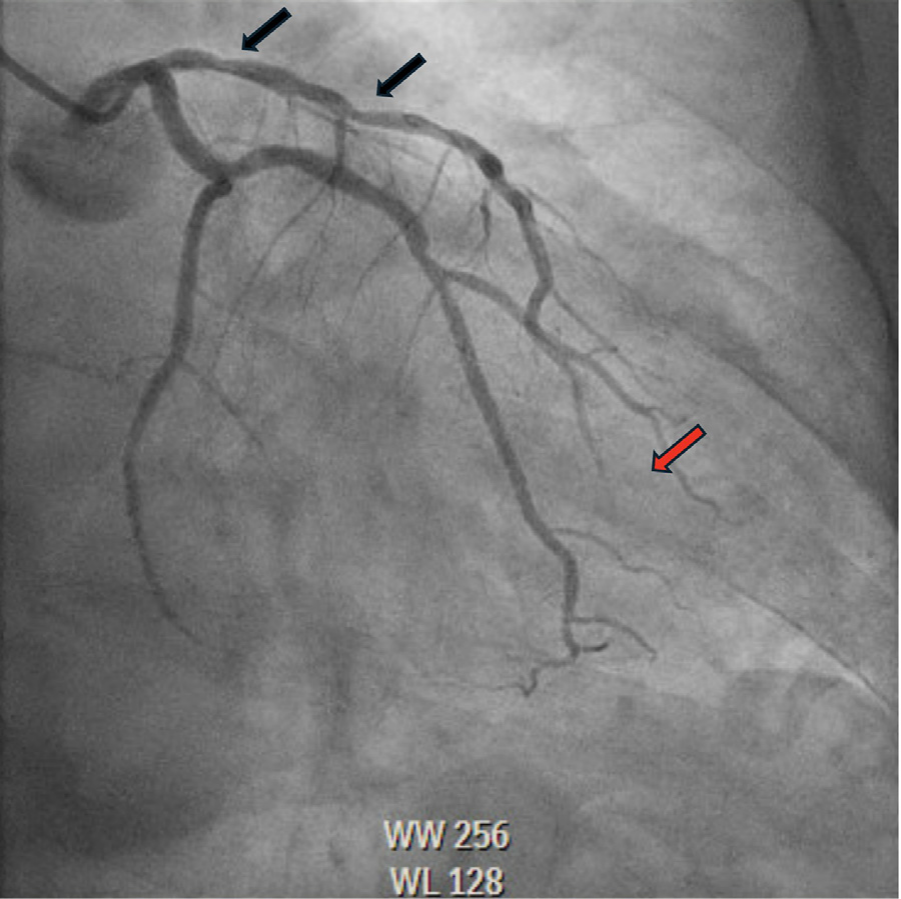
Coronary angiography showing severe stenosis of the proximal and mid left anterior descending artery (LAD) (Black arrows) prior to stent deployment without distal filling of the LAD (Red arrow).

**Fig. 2:**
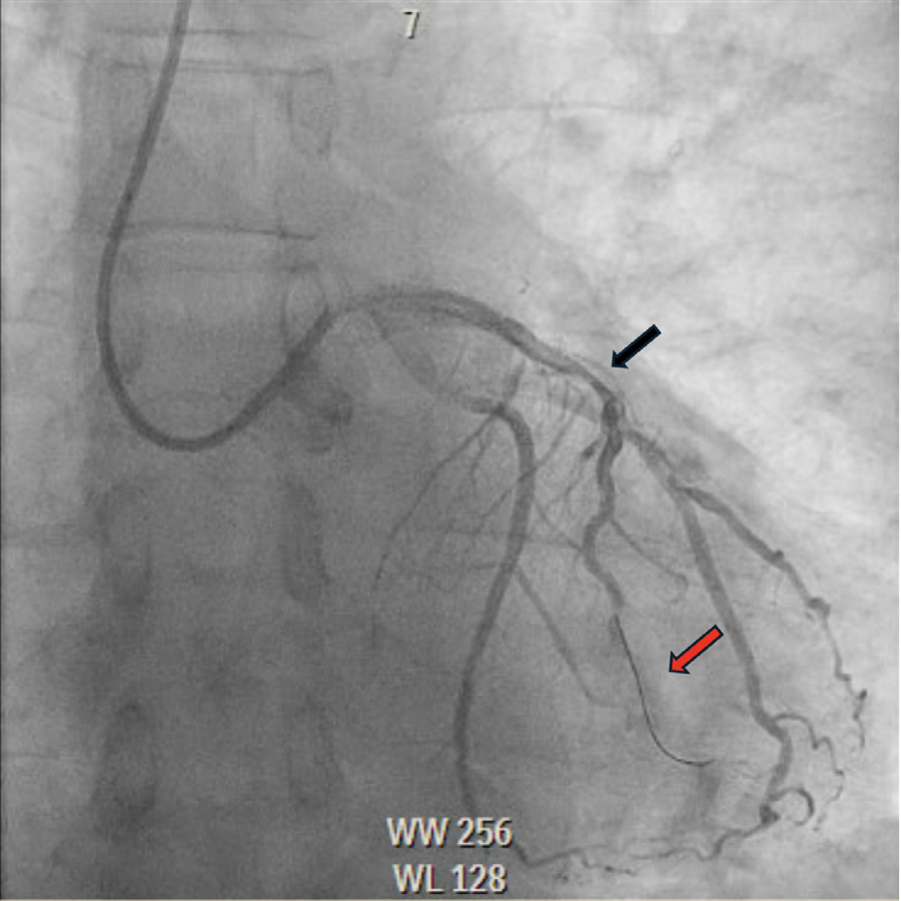
Coronary angiography showing coronary vasospasm of the mid LAD (Black arrows) after optical coherence tomography (OCT) catheter has been withdrawn without distal filling of the LAD (Red arrow).

**Fig. 3:**
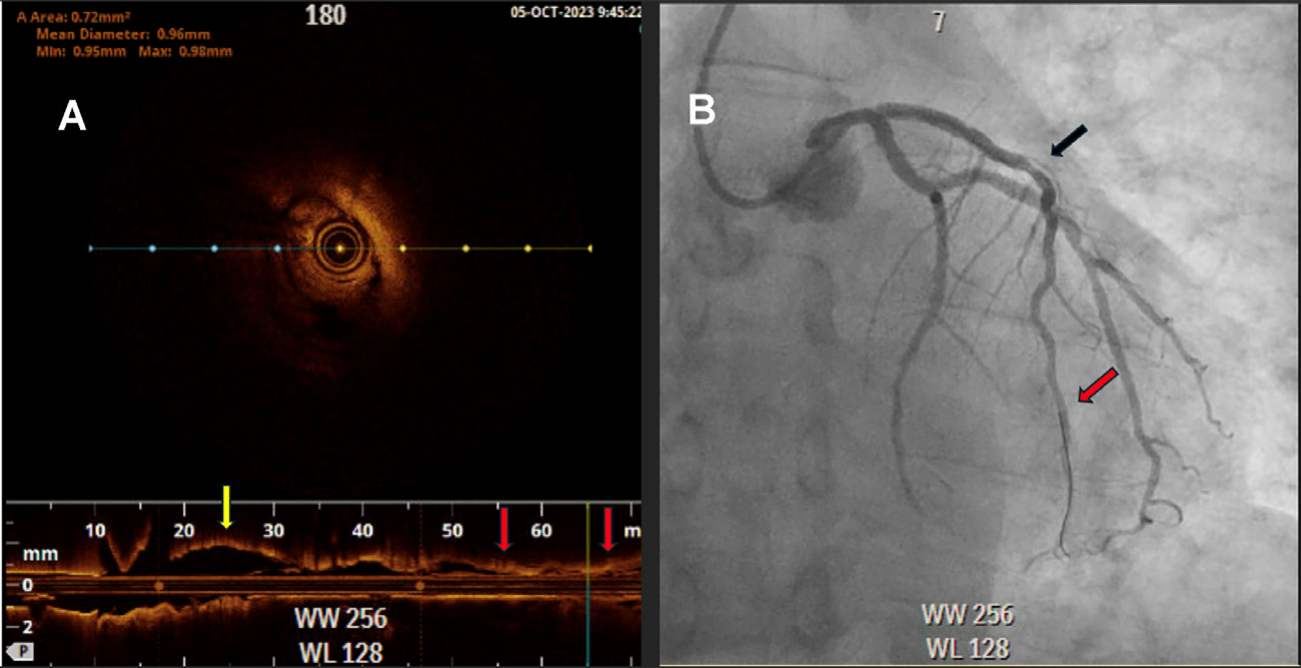
(A) OCT imaging of the LAD showing proximal portion without spasm (yellow arrow) along with coronary vasospasm (red arrows) which occurred following placement of the OCT catheter. A cross-sectional view of the spasmed artery is seen in the center of frame, (B) Coronary angiography post stent deployment and intracoronary nitroglycerin with resolution of coronary vasospasm (Black arrow) and distal filling of the LAD (Red arrow).

A 57-year-old male with past medical history significant for primary hypertension, pulmonary hypertension, ongoing tobacco abuse (1 pack per day of cigarettes) and mixed hyperlipidemia who had presented to the hospital with worsening substernal chest pain that was not associated with activity. He was treated medically with Amlodipine 5 mg daily, Atorvastatin 40 mg daily, Ranolazine 500 mg twice daily without improvement in symptoms. He was not treated with beta blockade due to baseline resting sinus bradycardia. He was brought to the cardiac catheterization laboratory for left coronary angiography to further evaluate the etiology of his chest pain. During coronary angiography a 70% lesion was noted in the proximal segment of the left anterior descending (LAD), followed by a 70% lesion in the mid segment (Fig. 1). Fractional Flow Reserve (FFR) was performed across the lesions with a result of 0.78 which was physiologically flow-limiting. A 3.0 × 20 mm balloon was advanced into the LAD, with multiple inflations performed through the proximal and mid LAD. A 3.25 × 38 mm drug-eluting stent was then deployed in the proximal to mid segments of the LAD, with OCT performed to evaluate strut apposition which revealed under expansion of the stent. A 3.5 × 50 mm noncompliant balloon was used for final expansion. Angiography postexpansion revealed a patent stent in the proximal to mid LAD without evidence of spasm (Fig. 2). OCT was reinserted and revealed a well-expanded and well apposed stent. Following OCT, spasm was noted in the LAD distal to the stent, this resolved with administration of 300 mcg of intracoronary nitroglycerin (Figs. 3A and B). There were no further complications during the procedure.

## Discussion

OCT is a technology that was first introduced in 1991 that uses the coherence of light to provide cross-sectional imaging of biological tissues by measuring optical reflections [1]. It has a high rate of reproducibility and low levels of energy used in the range of 5.0-8.0 mW, below what causes damage to tissue [6,7]. In one series of 468 patients, none had coronary artery vasospasm during OCT [7]. OCT has been used to differentiate the presence of coronary artery vasospasm versus atherosclerotic disease [7,8]. Catheter-induced vasospasm is a rarely reported complication. Studies are warranted to investigate whether there is some underlying genetic pathophysiology. Proposed mechanisms of catheter-induced coronary artery vasospasm include mechanical irritation, dysfunction of the endothelium, hyperactivity of the smooth muscles, and activation of cardiac stress receptors. Retrospective studies have showed that vast majority of catheter-related coronary spasms tend to occur in the right coronary artery (RCA) and majority of the patients also have concurrent vasospastic angina [2]. Sueda *et al.* conducted a multivariable analysis and found that a positive vasospasm test, young patients and diabetes were strong determinants for catheter-induced vasospasm. Their review also concluded that characteristics relating to the type and size of catheter used was not associated with catheter-induced vasospasms and interventionalists should rely more on the patient's clinical characteristics to predict this rare occurrence. History of tobacco use has been also reported as a risk for development of coronary vasosospasm in general [9]. Review of literature has also shown that patients who have a history of coronary artery spasms and Prinzmetal's angina are more likely to develop catheter-induced vasospasm therefore the use of calcium channel blocking agents and nitrate therapy has been proposed to be beneficial to these individuals [10]. Interventionalists need to be cognizant of coronary vasospasm from OCT catheters to avoid stenting of coronary arteries angiographically free of stenosis when not indicated. Intracoronary administration of nitroglycerine with OCT imaging is of utility when this occurs to best differentiate vasospasm secondary to different etiologies. A transthoracic echocardiogram can also help assess wall motion abnormalities from ischemic cardiomyopathy if present. The use of cardiac MRI for predicting long-term outcomes of OCT catheter-induced vasospasm needs to be investigated with studies in the future. Prospective studies are warranted to best identify what therapies can provide optimal care when OCT catheter-induced vasospasm is encountered.

## Conclusion

OCT catheter-induced coronary artery vasospasm is a rare complication which most commonly occurs in the RCA, young, patients with diabetes, and tobacco users at highest risk. In our case, introduction of the OCT catheter caused vasospasm of the LAD, which resolved with administration of nitroglycerine. There is yet to be published case reports demonstrating coronary artery vasospasm in the LAD from this cause. This suggests further studies may reveal a heretofore unreported possible complication of OCT.

## Author contributions

FM, ZAK, MM, EDG contributed to writing the original draft. CS, SH, SS, AA, JDD, MAW, MSA, MK, RR contributed to reviewing and editing the manuscript for important intellectual content and approval of the final manuscript.

## Patient consent

Written, informed consent for publication of their case was obtained from the patient.
